# 

**Published:** 1857-10

**Authors:** 


### CONTENTS OF THE OCTOBER NUMBER.

ORIGINAL COMMUNICATIONS. Page.

ART. I.—Fractures of the Malar Bone, Superior Maxillary, Ethmoid, and of other bones of the Face. By Frank H. Hamilton, M. D., - - - - - - - - - - - - - 257

ART. II.—Abstract of the Proceedings of the Buffalo Medical Association, - - - - - - 268

ART. III.—A Case of Accidental Swallowing of a Cent of the old coinage. Communicated to the Buffalo Med. Association. By P. H. Strong, M. D., Buffalo, - - - - - - - 288

ART. IV.—Reports of Cases occurring in the Medical Ward of the Buffalo Hospital. Reported by Dr. A. W. Nichols, - - - - - - - - - - - - - - - - - - - - 291

ART. V.—A Case of Urticaria, induced by the Ingestion of the Allium Porrum. By William G. Meachem, M. D., Warsaw, N. Y., - - - - - - - - - - - - - - 297

ART. VIII.—Monthly Record of Deaths in the City of Buffalo. By C. L. Dayton, M.D., Health Physician, - - - - - - - - - - - - - - - - - - - - - 302

EDITORIAL DEPARTMENT.

Obituary of Dr. Marshall Hall, - - - - - - - - - - - - - - - 304

Dr. Ch. Robin, of Paris, - - - - - - - - - - - - - - - - - 313

Dr. Fell the American Cancer Doctor, - - - - - - - - - - - - - 313

Fat Men, - - - - - - - - - - - - - - - - - - - - - - 314

University of Buffalo. - - - - - - - - - - - - - - - - - - 315

